# *Abiotrophia defectiva* liver abscess in a teenage boy after a supposedly mild blunt abdominal trauma: a case report

**DOI:** 10.1186/s12876-020-01409-6

**Published:** 2020-08-14

**Authors:** Petar Rasic, Srdjan Bosnic, Zorica V. Vasiljevic, Slavisa M. Djuricic, Vesna Topic, Maja Milickovic, Djordje Savic

**Affiliations:** 1 Department of Abdominal Surgery, Mother and Child Health Care Institute of Serbia “Dr. Vukan Cupic”, Radoja Dakica 6-8, Belgrade, Serbia; 2 Department of Clinical Microbiology, Mother and Child Health Care Institute of Serbia “Dr. Vukan Cupic”, Radoja Dakica 6-8, Belgrade, Serbia; 3 Department of Clinical Pathology, Mother and Child Health Care Institute of Serbia “Dr. Vukan Cupic”, Radoja Dakica 6-8, Belgrade, Serbia; 4 Banjaluka University School of Medicine, Banjaluka, Bosnia and Herzegovina; 5 Department of Radiology, Mother and Child Health Care Institute of Serbia “Dr. Vukan Cupic”, Radoja Dakica 6-8, Belgrade, Serbia; 6 School of Medicine, University of Belgrade, Belgrade, Serbia

**Keywords:** Liver abscess, *Abiotrophia defectiva*, Liver trauma, Children, Case report

## Abstract

**Background:**

A pyogenic liver abscess (PLA) represents a pus-filled cavity within the liver parenchyma caused by the invasion and multiplication of bacteria. The most common offender isolated from the PLA in children is *Staphylococcus aureus. Abiotrophia defectiva* is a Gram-positive pleomorphic bacterium, commonly found in the oral cavity, intestinal, and genitourinary mucosa as part of the normal microbiota. It has been proven to be an etiological factor in various infections, but rarely in cases of PLA. The case presented here is, to the best of our knowledge, the first pediatric case of PLA caused by *A. defectiva*.

**Case presentation:**

A 13-year-old Caucasian boy presented with a two-day history of abdominal pain, fever up to 40 °C, and polyuria. Contrast-enhanced computed tomography (CT) scan revealed a single, multiloculated liver lesion, suggestive of a liver abscess. The boy had sustained a bicycle handlebar injury to his upper abdomen 3 weeks before the symptoms appeared and had been completely asymptomatic until 2 days before admission. He was successfully treated with antibiotic therapy and open surgical drainage. *A. defectiva* was isolated from the abscess material. Histopathology report described the lesion as a chronic PLA.

**Conclusions:**

*A. defectiva* is a highly uncommon cause of liver abscess in children. In such cases, various predisposing factors should be considered, including antecedent blunt abdominal trauma.

## Background

A liver abscess (LA) represents a pus-filled cavity within the liver parenchyma caused by the invasion and multiplication of microorganisms. The etiology of LA may be bacterial, parasitic (amebic, mostly), mixed (pyogenic superinfection of a parasitic abscess) or, more rarely, fungal. Bacterial infection represents the most common cause of hepatic abscess in developed countries, giving rise to a pyogenic liver abscess (PLA) [1]. Previously, it had been shown that a wide diversity of bacteria was associated with this kind of hepatic lesion [2–4]. In Central Europe, PLA is most frequently caused by *Escherichia coli, Streptococcus* spp., and *Staphylococcus* spp., whereas *Klebsiella pneumoniae* causes the majority of PLA cases in Southeast Asia [2]. The most common offender isolated from the PLA in children is *Staphylococcus aureus* [3]. Anaerobes, microaerophilic streptococci, and Gram-negative rods (such as *E. coli* and *Klebsiella* spp.) are also frequently recognized as causative organisms of PLA in the pediatric population [4].

The bacteria can reach the liver parenchyma in different ways: through biliary ducts (i.e., chole (docho) lithiasis, obstructing tumor, stricture or congenital anomaly of the biliary tree), by portal vein seeding (in case of appendicitis, diverticulitis, inflammatory bowel disease or postoperative intra-abdominal infections), by hepatic artery seeding (due to the hepatic artery thrombosis/chemoembolization or bacteremia), through direct extension of bacteria (from a subphrenic abscess, perinephric abscess or cholecystitis), or by penetrating trauma. However, according to some studies, in 18–66% of cases, PLA is cryptogenic (with no obvious underlying cause) [5].

Here, we present a case of a teenage boy with a pyogenic liver abscess caused by *Abiotrophia defectiva,* which is, to the best of our knowledge, the first reported pediatric case of PLA caused by this organism. The potential pathogenetic role of antecedent asymptomatic blunt liver trauma (BLT) is discussed.

## Case presentation

**Fig. 1:**
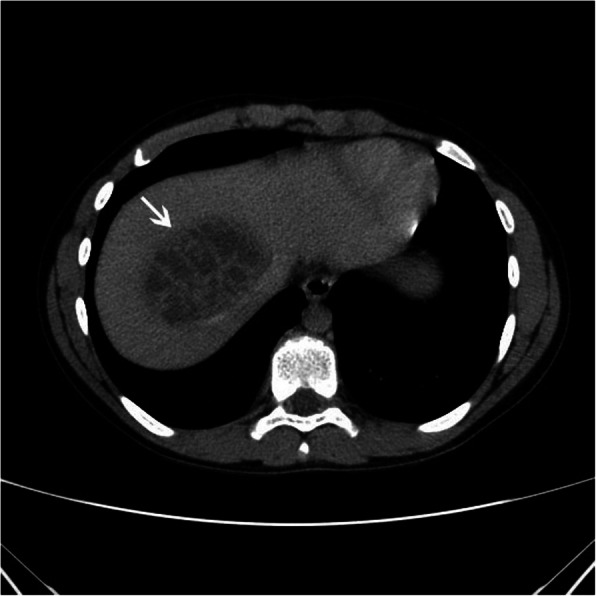
Axial contrast-enhanced portal phase CT image shows a single, low-attenuation, multilocular lesion in the segment VIII of the liver

A 13-year-old Caucasian boy presented to the regional hospital with a two-day history of abdominal pain, fever up to 40 °C, and polyuria. Three weeks before the symptoms appeared, the boy had fallen off his bicycle and sustained a handlebar injury to his upper abdomen. However, he was completely asymptomatic in the meantime and, when asked about the possibility of a recent abdominal trauma, the child and his mother at first denied any such event. Apart from that, his general past medical history was unremarkable. Physical examination on admission revealed a soft, non-tender, and non-distended abdomen, without masses. The liver and spleen were not palpable below the costal margins. The laboratory tests showed significantly elevated inflammatory parameters (C-reactive protein (CRP) of 154.68 mg/L and erythrocyte sedimentation rate of 53 mm/h), leukocytosis (his white blood cell count was 13.81 × 10^9^/L), and mildly elevated levels of aminotransferases (alanine aminotransferase (ALT) and aspartate aminotransferase (AST) levels were 77 IU/L and 52 IU/L, respectively). The urinalysis did not detect the presence of nitrites and white blood cells, and his urine culture was negative. Abdominal ultrasound revealed a unifocal, predominantly hypoechoic lesion in the right hepatic lobe, with irregular internal echoes. Contrast-enhanced portal phase computed tomography (CT) scan showed a single, well-defined, low-attenuation liver lesion, located in the segment VIII, measuring 65x56x41mm (LLxAPxCC) (Fig. 1). The lesion was multiloculated, with contrast-enhanced internal septa, which represents a “cluster sign” of PLA. Right and middle hepatic veins were compressed and slightly dislocated by lesion, but patent. Empiric antibiotic therapy was initiated with ceftriaxone, and subsequently, amoxicillin/clavulanate was added. Enzyme-linked immunosorbent assay (ELISA) for the determination of anti-*Echinococcus* IgG antibodies was negative. On the fourth day after admission, the boy’s overall condition significantly improved, and he was afebrile for the rest of his hospital stay in the regional hospital.

On day 7 after admission, the patient was transferred to the tertiary care pediatric hospital for further diagnostic evaluation and treatment. The physical examination on admission was unremarkable; laboratory test values were within normal ranges except for leukocytosis with neutrophilia (total leukocyte count: 13.35 × 10^9^/L; neutrophils: 74.42%), and elevated levels of CRP (49.8 mg/L) and ALT (72 IU/L). Alpha-fetoprotein and beta-human chorionic gonadotropin values were within the reference ranges. The patient’s blood culture showed no growth.

**Fig. 2:**
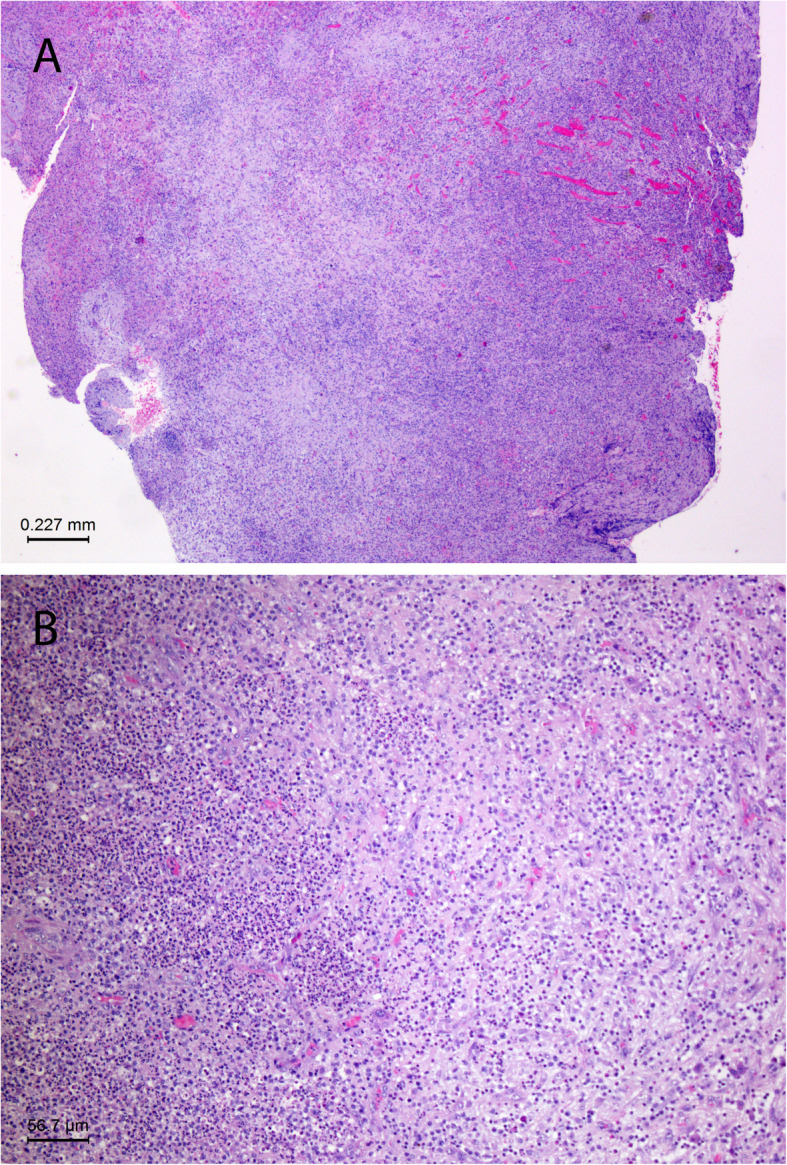
**a** From right to left, typical layers of the wall of a chronic PLA are visible: liquefaction necrosis with the abundance of neutrophils, granulation tissue with congested, young capillaries, a thick zone of proliferating fibroblasts and a variably thick layer of preserved liver parenchyma at the periphery of the specimen. (HE, × 25). **b** The inner layer of the chronic pyogenic abscess. To the right of a zone of neutrophil-rich liquefaction necrosis is a thick layer of granulation tissue with numerous, young capillaries and initial fibroblastic proliferation. (HE, × 100)

Antibiotic therapy was switched to cefazolin and metronidazole. On the 11th hospital day, open surgical drainage of the liver abscess was performed. Upon entering the abdominal cavity through the right subcostal incision, the falciform ligament, the coronal ligament and the right triangular ligament were sectioned to allow the mobilization of the right lobe of liver, and a solitary lesion was identified in its anterosuperior portion. The incision on the surface of the liver parenchyma allowed access to the multiloculated hepatic abscess cavity filled with viscous pus. The loculations were manually broken down to ensure complete drainage and, additionally, a surgical drain was left in situ. The abscess material was sent for microbiological analysis. Gram staining revealed Gram-positive pleomorphic cocci and coccobacilli arranged in chains. Cultures yielded scanty growth on Columbia and chocolate agar. MALDI-TOF MS identified the isolated bacterium as *Abiotrophia defectiva.* Also, a tissue sample of six fragments measuring 2x2x0.5 cm in total was sent for histopathological analysis. Microscopically, around a central area of the confluent microabscesses, a thick zone of granulation tissue with hyperemic, young capillaries, and peripheral maturation in the form of proliferating fibroblasts, presented the typical picture of chronic PLA (Fig. 2). The surrounding hepatocytes showed mild regenerative atypia.

Postoperatively, the patient received triple-antibiotic therapy with cefazolin, gentamicin, and metronidazole. After the microbiological culture report came in, these were switched to ceftriaxone. The boy recovered very well after the surgical procedure. He spent 3 weeks in total on intravenous antibiotic therapy, followed by 3 weeks of peroral amoxicillin/clavulanate. Meanwhile, a thorough oral cavity and dental examination was performed to look for a potential source of infection, and no decayed teeth or pathological changes were identified. The patient was doing well after 3 months of follow-up. His physical examination and laboratory tests showed no abnormalities, and the abdominal ultrasound imaging indicated that pyogenic liver abscess had resolved to almost normal liver parenchyma.

## Discussion and conclusions

During the first three decades of the twentieth century, the most frequent cause of PLA was pylephlebitis secondary to appendicitis [6]. Nowadays, the incidence of this complication is significantly reduced because of the expeditious surgical treatment of intra-abdominal infections and the advances made in antimicrobial therapy [7]. Currently, biliary tract disease is the most common identifiable cause of PLA in adults [8].

In the pediatric population, many predisposing factors may be associated with the development of LA, namely, congenital immunodeficiency disorders (chronic granulomatous disease, Papillon-Lefèvre syndrome, deficiency of C1 complement component, hyperimmunoglobulin E syndrome), acquired immunosuppression, protein-calorie malnutrition, sickle cell disease, helminthic infections, congenital biliary tree anomalies, and trauma [3, 4].

Penetrating trauma may cause the development of PLA by inoculating bacteria directly into the liver parenchyma. On the other hand, BLT may provide conditions for the proliferation of microorganisms due to localized hepatic necrosis, intrahepatic hemorrhage, and bile leakage, which may lead to abscess formation [3, 4, 9]. Liver trauma, including penetrating and blunt injury, is responsible for less than 5% of all PLA cases [9]. Moreover, PLA is a known complication of the non-operative treatment of BLT, although it occurs quite infrequently – in about 0.7% of patients, according to combined results of three large studies [10]. In the series of Hsieh et al., which included 395 patients with BLT who were treated non-operatively, this complication was identified slightly more frequently, in 1.5% of cases. All of these patients suffered from liver injury grade 3 or 4 according to the American Association for the Surgery of Trauma Hepatic Injury Scale (1994 revision) [9, 11]. Notwithstanding, Fabian et al. reported that perihepatic abscess formation was observed even in patients with liver trauma grade 1 or 2 [12]. However, this study did not specify the precise location of the abscesses (intrahepatic, subhepatic or subphrenic) nor whether they were the complication of blunt or penetrating liver trauma. Although the anamnestic data suggested that our patient had suffered an apparently mild, symptom-free, blunt abdominal trauma, and no other liver lesions were seen on the CT scan performed 3 weeks after that event, we cannot exclude its potential pathogenetic role in this case.

It is plausible to speculate that oxygen depletion and blood in post-traumatic hematoma provided fertile ground for oral bacteria seeded through transient bacteremia. However, the oral cavity and dental examination showed no abnormalities in our patient. On the other hand, it is well-known that asymptomatic bacteremia can occur in normal daily activities, such as conducting oral hygiene. In a healthy person, these clinically benign infections are transient and cause no further sequelae [13]. Alternatively, the causative role of bacterial invasion originating from an unidentified gastrointestinal injury following blunt abdominal trauma in developing PLA has been considered by some authors [12]. Therefore, the exact route of entry of bacteria cannot be assuredly determined in our patient.

The percutaneous drainage in combination with intravenous antibiotics is currently the standard treatment for the majority of PLA. However, according to the current practices, if the abscess is multiloculated, has a diameter of > 5 cm, or is located in an anatomical area that is difficult to access percutaneously, open surgical drainage should be considered [14, 15]. PLA in our patient met all these criteria, so we opted for the open surgical drainage.

*A. defectiva* is a pleomorphic bacterium first described in 1961 and was previously referred to as nutritionally variant streptococci (NVS) [16]. NVS are fastidious Gram-positive bacteria, requiring either L-cysteine- or pyridoxal-supplemented medium to support growth. DNA-based technology allowed reclassification of the organisms that previously belonged to NVS in two genera: *Abiotrophia* and *Granulicatella* [17]. The genus *Abiotrophia* currently includes a single species, *A. defectiva* [18]. *A. defectiva* is commonly found in the oral cavity, intestinal, and genitourinary mucosa as a part of the normal microbiota [19], but also has been proven to be an etiological factor in various infections. The most frequent is endocarditis, with more than 125 cases reported in the English literature [20]. Furthermore, there are published cases of other *A. defectiva* infections: ocular infections, meningitis, peritoneal dialysis-related peritonitis, pancreatic abscess, prosthetic joint infections, otitis media, and osteomyelitis [19, 21]. Nevertheless, *A. defectiva* has been rarely isolated in patients with PLA.

In the study of Reyna-Fabián et al., which investigated the difference in bacterial diversity in the amebic and bacterial liver abscess, *Abiotrophia* genome was detected in the abscess aspirate of one patient who was clinically diagnosed with amebic liver abscess. This patient had a positive ELISA test for anti-amebic antibodies. Apart from *Abiotrophia*, DNA analysis of the abscess material detected other bacterial species, including *Granullicatela* and *Streptococcus* sp., but none of the *Entamoeba* molecular markers were detected [22]. Also, Cavrić et al. reported a case of a 78-year-old patient with a polymicrobial liver abscess caused by *Actinomyces odontolyticus, Abiotrophia* sp.*, Haemophilus parainfluenzae, Streptococcus intermedius,* and *Streptococcus anginosus*, probably related to subacute cholecystitis [23].

The histopathology report described the lesion as a chronic PLA. Albeit, its clinical presentation with sudden onset of high-grade fever and abdominal pain, as well as elevated CRP level, was more similar to the acute PLA, which is generally caused by more virulent microorganisms such as *S. aureus* and *E. coli*. We may hypothesize that, due to its low-virulence potential, *A. defectiva* did not evoke a vigorous systemic inflammatory response in our patient. Therefore, the preclinical phase of the disease was quite insidious, and only the advancement of liver damage triggered the onset of acute inflammation. Accordingly, a long preclinical phase allowed for significant enlargement of the abscess, leading to difficulties in management and requiring the use of a more invasive surgical approach.

To conclude, *A. defectiva* is, due to its low virulence, an uncommon cause of liver abscess in healthy individuals. In such cases, various predisposing factors should be considered, such as previous liver disease, immunodeficiency, and trauma. In our patient, a possible explanation might be that the neglected blunt hepatic trauma provided a habitat for the proliferation of this microorganism within the liver parenchyma. Nevertheless, the cryptogenic origin of the abscess cannot be excluded.

On the other hand, we may argue that our case is the first well-documented case of *A. defectiva-*caused bacterial hepatic abscess in a pediatric patient. However, having in mind that our patient received multiple-antibiotic therapy before sampling for microbiological culture, it is unclear whether *A. defectiva* was the sole causative agent or this might have been a case of primarily polymicrobial infection.

## Data Availability

The authors declare that all data supporting the findings of this case report are available within the article.

## Abbreviations

ALT: Alanine aminotransferase
AST: Aspartate aminotransferase
BLT: Blunt liver trauma
CRP: C-reactive protein
CT: Computed tomography
ELISA: Enzyme-linked immunosorbent assay
LA: Liver abscess
NVS: Nutritionally variant streptococci
PLA: Pyogenic liver abscess
